# Recovery of organic waste from a wastewater treatment plant, improved with plant growth promoting bacteria: model of *Quercus suber* L.

**DOI:** 10.3389/fmicb.2026.1754063

**Published:** 2026-05-29

**Authors:** Daniel González-Reguero, Marina Robas-Mora, Efren García Ordiales, Vanesa M. Fernández-Pastrana, Diana Penalba-Iglesias, Agustín Probanza Lobo, Pedro A. Jiménez Gómez

**Affiliations:** 1 Department of Pharmaceutical Science and Health, CEU San Pablo University, Madrid, Spain; 2 Department of Mining Exploitation and Prospecting, School of Mining, Energy and Materials Engineering, University of Oviedo, Oviedo, Spain

**Keywords:** antibiotic resistance, *Bacillus pretiosus*, biofertilizers, cenoantibiogram, PGPB, *Pseudomonas agronomica*, *Quercus suber*, valorisation

## Abstract

Cork oaks (*Quercus suber* L.) are key tree species in Mediterranean ecosystems, playing a crucial role in fire mitigation due to their thick, fire‑resistant bark, while also contributing to biodiversity conservation and soil stability. Integrating waste valorization strategies with biofertilizers based on plant growth‑promoting bacteria (PGPB) may enhance reforestation efficiency. This study evaluated different irrigation regimes under controlled phytotron conditions, including water, organic fertilizer derived from a wastewater treatment plant (WWTP), and sterilized WWTP fertilizer, combined with *Bacillus pretiosus* CECT30673^T^ and *Pseudomonas agronomica* CECT30673^T^. Microbial functional diversity (Shannon index), antibiotic resistance profiles, and rhizosphere community structure were assessed using 16S rRNA‑based metagenomic analyses, including taxonomic composition, beta diversity, and genus‑level relative abundances. Plant performance was evaluated through biomass production, stem length, and nutritional parameters, including protein composition, sugar content, and fatty acid profile. The application of PGPBs together with WWTP‑derived fertilizers resulted in a significant increase in plant biomass and stem length compared to traditional water irrigation. Nutritional quality was also significantly improved, with higher protein, sugar, and fatty acid contents. Additionally, the combined treatments reduced minimum inhibitory concentrations (MICs) within the rhizosphere microbial community while maintaining its functional and structural stability. These results demonstrate that combining PGPBs with WWTP‑derived matrices enhances cork oak growth and nutritional quality without disrupting native soil microbiomes, supporting their potential as sustainable tools for Mediterranean reforestation.

## 1 Introduction

Global forestry restoration faces unprecedented challenges due to accelerating land degradation and wildfires, accounting for a net loss of approximately 200 Mha of forest cover between 1990 and 2020 (FAO, 2021). Traditional restoration has relied on conventional chemical fertilization; however, its efficacy is constrained by environmental costs and an inability to restore soil biological functions. In a context of increasing global population pressure, the United Nations 2030 Agenda (Goal 12: Responsible Consumption and Production) underscores the need for sustainable resource management and waste recovery. Consequently, there is an urgent need to shift toward active microbiome engineering through the reuse of industrial byproducts.

The valorisation of sludge, a mixture of water and flocculated organic matter from wastewater treatment plants (WWTPs, hereafter referred to as EDAR following Spanish regulatory nomenclature), presents a significant opportunity to reintegrate waste streams into productive use. In accordance with Law 7/2022, of 8 April (BOE, 2022) and the National Integrated Waste Plan, WWTPs must ensure correct management to protect human health and the environment. While this sludge consists of solids separated through natural or artificial processes (Basar et al., 2024), it cannot be applied directly as a soil amendment without prior physicochemical or biological treatment, owing to its pathogen load and trace contaminant content. However, it serves as a substrate for Plant Growth-Promoting Bacteria (PGPB), which mineralize complex organic compounds into plant-available forms (González et al., 2021; Nosheen et al., 2021). These bacteria enhance soil fertility sustainably by fixing atmospheric nitrogen, producing phytohormones, and suppressing soil-borne pathogens, thereby reducing dependence on synthetic chemical inputs (Lorenz et al., 2025; Rotoni et al., 2025).

This biotechnological approach is vital for the cork oak (*Quercus suber* L.), a sclerophyllous Fagaceae native to the western Mediterranean, where it occupies over two million hectares (Villada et al., 2018; Sousa et al., 2022). As a slow-growing species characteristic of pastureland landscapes, it provides shade and reduces soil temperature, thereby facilitating understory growth and ecosystem regeneration (de Viñas et al., 2003). Evolutionarily, its thick corky bark confers fire resistance (Molina et al., 2018), supporting biodiversity, CO₂ sequestration, and genetic diversity (Lorenzo et al., 2009; Bugalho et al., 2011; Rives et al., 2012). Spain is the world’s second-largest producer of cork (31% of global production), according to the Natural Cork Council. In 2023, cork extraction reached 62,000 tonnes; official annual production typically ranges from 49,000 to 70,000 tonnes, with an additional estimated 30,000 tonnes from private forests in Andalusia (Ros et al., 2024).

The novelty of this study lies in the high-resolution assessment of these biofertilizers within a One Health framework. While PGPB improve soil quality and aid in decontamination (González-Reguero et al., 2022, 2023), their environmental release requires careful biosafety evaluation. Soil represents a major environmental reservoir of antibiotic resistance genes (ARGs), which can disseminate through horizontal gene transfer among bacterial communities. Thus, valorisation strategies must incorporate monitorization of both physicochemical soil parameters and shifts in the resident microbiological communities (Hernando-Amado et al., 2019; Gufe et al., 2025).

To address this, we employed metagenomic and 16S rRNA-based analyses complementing culture-dependent approaches with culture-independent methods to provide a more comprehensive characterization of microbial genetic potential (Nwachukwu and Babalola, 2022; Yousuf et al., 2025). This study investigated the impact of chemical fertigation supplemented with two PGPB strains—*Bacillus pretiosus* (C1) and *Pseudomonas agronomica* CECT30673T (C2)—on the metabolic diversity of soils associated with *Q. suber* seedlings. By integrating Minimum Inhibitory Concentrations (MICs) with compositional profiling, we evaluated the persistence of introduced strains and their broader implications for ecosystem resilience and the “resistome” of Mediterranean forestry systems (Li et al., 2024a).

## 2 Materials and methods

### 2.1 Bacterial strains

The bacterial strains tested were isolated from the rhizosphere of *Medicago sativa*. These strains have been described and characterized by the “Environmental Bacterial Microbiology” (GIR-MICROAMB) research team of the Faculty of Pharmacy of the San Pablo CEU University. These strains are of biotechnological interest owing to their plant growth-promoting characteristics (Table 1). Neither transmissible antibiotic resistance genes nor virulence determinants have been detected in these strains (Robas Mora et al., 2023).

**Table 1:** PGPB characteristics of the tested strains.

Identification (WGS)	IAA (μg.mL^10−1^)	ACCd (p/a)	Siderophores (p/a)
*Bacillus pretiosus* CECT30674^T^	5.61 ± 0.03	−	+
*Pseudomonas agronomica* CECT30673^T^	5.85 ± 0.09	+	+

WGS (whole genome sequence), IAA (production of 3-indoleacetic acid), ACCd (production of 1-aminocyclopopane-1-carboxyl deaminase) and p/a (presence (+)/absence).

### 2.2 Tested plants and substrate

The biological tests were carried out under controlled laboratory conditions on seedlings of two cork oak saps, *Q. suber* L., supplied by IMIDRA (Madrid Institute for Rural, Agrarian and Food Research and Development). These seedlings were derived from native plant seeds in the Community of Madrid, whose purpose is to repopulate forests to avoid genetic contamination. For the growth test under laboratory conditions, 10 cm × 8 cm seedbeds with 35 pots of 18 cm high were used, placed in forest or leachate trays. For each treatment 35 seedlings (replicates) grown in pot were used. Three irrigation matrices (chemical treatment) were tested: water (W), organic waste from a WWTP and the same sterilized waste (EDAR_ST). Each of them was tested with three biological treatments: control without inoculum (C0), supplemented with *B. pretiosus* CECT30674^T^ (C1) and supplemented with *P. agronomica* CECT30673^T^ (C2).

### 2.3 Preparation of bacterial suspensions and biofertilizer

#### 2.3.1 Preparation of the irrigation chemical matrix

The WWTP residue (Industrias Cárnicas Villar, S.A., Soria, Spain) was used in a dilution of 1/512 (V_WTTP_/V_H2O_). For all the trials in this work, this dilution was used as the basis for the formulation of the biofertilizer (incorporation of inoculum). The physicochemical composition of the WWTP is presented in Table 2. The data showed remarkable values for conductivity at 25 °C of 1,612 μS.cm^−1^, with a biochemical oxygen demand (BOD5) of 3,200 mg O₂.L^−1^ and a chemical oxygen demand (COD) of 6,720 mg O₂.L^−1^. Kjeldahl nitrogen was measured at 296.7 mg.L^−1^, and the pH of the residue was 6.5. Suspended solids reached 3,228 mg.L^−1^, while potassium and orthophosphates were recorded at 60.2 mg.L^−1^ and 74.32 mg PO₄.L^−1^, respectively. In addition, the concentration of sulfates was 89.0 mg.L^−1^. This data indicated a composition rich in essential nutrients and chemical elements that could have significant effects on biofertilization and soil improvement. To eliminate the microbiota of the WWTP organic waste, an aliquot of the same (EDAR_ST) was sterilized by autoclaving at 121 °C for 20 min at 1 atmosphere of pressure.

**Table 2:** Physicochemical composition of the WWTP sludge (analysis carried out by LabAqua. Tests covered by ENAC accreditation n°109/LE 28).

	Parameters	Methods	Results	Units
Physico-chemical characteristics	Conductivity at 20 °C	A-F-PE-0015 Electrometry	1,454	μS/cm
	Conductivity at 25°C	A-F-PE-0015 Electrometry	1,612	μS/cm
	Biochemical oxygen demand (BOD5)	A-F-PE-0002 Gauge method	3,200	mgO_2_/L
	Chemical oxygen demand	A-F-PE-0003 Digestion—Colorimetry	6,720	mgO_2_/L
	Chemical demand for decanted oxygen	A-F-PE-0003 Digestion—Colorimetry	4,220	mg/L
	Nitrates	A-F-PE-0010 Digestion	<0.05	mg/L
	nitrogen Kjeldhal	A-F-PE-0007 Kjeldhal	296.7	mg/L
	pH	A-F-PE-0010 Electrometer	6.5	U.pH.
	Suspended solids	A-F-PE-0006 Gravimetry	3,228	mg/L
	Toxicity	PIT-F/0012 Bioluminescence assay with *Vibrio fisheri*	14	U.T.
Majority cations	Potassium	A-D-PE-0025-ICP-OES	60.2	mg/L
Anions	Nitrates	A-BV-PE-0001 HPLC—Conductivity	<2.5	mg/L
	Orthophosphates	Ca-R-PE-0011 Spectrometry	74.32	mg PO_4_/L
	Sulphates	A-BV-PE-0001 HPLC—Conductivity	89.0	mg/L
	Sulphites	A-F-PE-0040 Volumetry	4.5	mg/L

#### 2.3.2 Biofertilizer preparation: addition of PGPBs to irrigation chemical matrices

First, bacterial suspensions of C1 (*B. pretiosus* CECT30674^T^) and C2 (*P. agronomica* CECT30673^T^) were prepared. To do this, they were grown in LB broth (Condalab^®^, Madrid, Spain) for 48 h (stirring 50 r.p.m., 25 °C ± 2 °C). The biofertilizer was prepared on a weekly basis to avoid non-specific transformations resulting from microbial metabolism (autochthonous microbiota and inoculum). Three liter of each treatment were prepared in each session and stored in refrigeration (4 °C). In order to standardize the microbial density to an equivalent of 0.5 McFarland (approximately 10^8^ cfu.mL^−1^), 100 mL of the bacteria grown in LB medium was added to 900 mL of the chemical irrigation matrix: water or the 1/512 dilution of WWTP/EDAR_ST, as appropriate and tested using UricultTM (Liofilchem srl, Italy).

#### 2.3.3 Growth test, irrigation protocol and plant growth conditions

The growth trial lasted 1 year and was carried out under controlled laboratory conditions in phytotron (photoperiod of 11 h of light and 13 h of darkness, light intensity of 505 μmol.m^−2^ s^−1^ (white and yellow light), temperature 18 °C ± 3 °C and relative humidity 30% ± 5%). The irrigation protocol was designed to emulate field conditions. It was carried out, with experimental volumes, in an aerial manner with 85 mL of each treatment, per alveolus, and 500 mL on the leachate tray. In this way, the relative humidity of the soil was guaranteed, without reaching saturation or waterlogging, which resulted in a weekly irrigation periodicity of 2 days.

### 2.4 Harvest, biomass measurement and nutritional analysis

The harvest was carried out after the trial sap was completed. It was destructive and involved the extraction of the aerial and radical part of each plant. Additionally, from the root part, the rhizospheric fraction of the soil (2 g per seedling) was obtained for the subsequent analysis of the edaphic communities, cenoantibiogram and metagenomics. For the calculation of the total biomass, the crop was left to dry at room temperature (22 °C ± 2 °C) and weighed after 1 week (dry weight, g). For nutritional analysis, the leaves were separated from the stem, and one measurement was performed per seedling. It was packaged and stored in refrigeration (4 °C), for shipment to the analysis laboratory (Rock River Labs Spain, Lalín, Pontevedra, Spain). The nutritional parameters that were measured were, both for the leaf and for the stem: total protein, amino acids, soluble protein, total available protein, acid-detergent fiber, amylase neutral-detergent fiber, ashless amylase neutral fiber, lignin, starch, water-soluble sugars, ethanol-soluble sugars, calcium, phosphorus, potassium, sulfur, total fatty acids, myristic acid, palmitic acid, stearic acid, oleic acid, linoleic acid, linolenic acid, acid-detergent fibre available at 240 h, non-digestible acid-detergent fibre rate at 240 h, acid-detergent fibre corresponding to organic matter at 240 h, acid-detergent fibre available in the total tract.

### 2.5 Rhizospheric microbial community extraction

For the extraction of the rhizospheric communities, the procedure described in García-Villaraco Velasco et al. (2009) was followed, as amended. To do this, 2 g of soil was suspended in 20 mL of sterile saline solution (0.45% NaCl) and homogenized with an Omni-Mixer homogenizer at 16,000 r.p.m. for 2 min. It was then centrifuged at 690 g for 5 min with a Hettich Zentrifugen centrifuge model Mikro 22R.

### 2.6 Analysis of soil microbial communities

#### 2.6.1 Functionality study: biolog ECO^®^

From the microbial suspension obtained and the dilutions selected according to the previous results, the 96 wells of the Biolog Eco plates^®^ (31 different carbon sources and one target (water), in triplicate) were loaded with 135 μL per well. The plates were incubated for 94 h at 25 °C, and their absorbance was measured at 595 nm every 24 h using the Asys UVM340 plate reading equipment and Micro WinTM V3.5 software. With the results of the absorbance measurements, the corrected absorbances were calculated by subtracting the value from the target and the mean of the corrected absorbance (AWCD) of all wells was calculated for each replicate. These data were used for the elaboration of kinetics, in order to detect the point (the day) when it entered stationary growth. At that time, AWCD data were used to calculate the metabolic (functional) diversity of the soil microbial community, using the Shannon–Weaver diversity index: H(m) = −∑ qi log2qi; where *n* is the corrected absorbance of each well and *N* is the total absorbance of all wells (Shannon, 1948).

#### 2.6.2 Study of community antibiotic resistance: cenoantibiogram

From the soil extract obtained in saline solution (NaCl 0.45%), it was verified that the density of viable microorganisms was >10^8^ F.C.U.·mL^−1^ (optical density (OD) = 0.5 McFarland). Mueller-Hinton agar (Condalab^®^, Madrid, Spain) was seeded and the MIC was evaluated using antibiotic Etest^®^ strips, in triplicate, for the following antibiotics: cefuroxime, cefuroxime axetil, cefoxitin, cefotaxime, ceftazidime, cefepime, ertapenem, imipenem, amikacin, gentamicin, nalidixic acid, ciprofloxacin, tigecycline, trimethoprim/sulfamethoxazole (BioMérieux^®^, Marcy l’Etoile, France). The plates were then incubated according to the manufacturer’s instructions (25 °C ± 2 °C). For the quantification of the minimum inhibitory concentration (MIC), the most restrictive halo was used as a reference (González-Reguero et al., 2023).

#### 2.6.3 Study of taxonomic diversity: metagenomics

**Figure 1:**
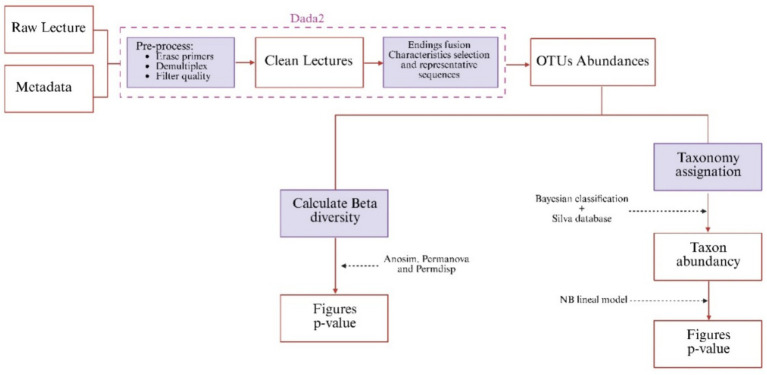
Sequence processing schema and analysis sequence.

The composition and structure of the bacterial communities present in the rhizosphere were evaluated by amplification and sequencing of the V3–V4 variable regions of the 16S rRNA gene, using the universal primers 341F (5′-CCTACGGGNGGCWGCAG-3′) and 806R (5′-GGACTACHVGGGTWTCTAAT-3′). PCR amplification was performed using 25 cycles under standard conditions. The metagenomic analysis included nine experimental treatments, each analysed in triplicate, resulting in a total of 27 biological rhizosphere soil samples. The experimental design comprised three substrate matrices: water (W), which served as the control matrix; wastewater residue (EDAR); and sterilised wastewater residue (EDARST). Each matrix was subjected to three bacterial conditions: no inoculation (C0), inoculation with bacterial strain C1, and inoculation with bacterial strain C2. This design generated the following nine treatments: WC0, WC1, WC2, EDARC0, EDARC1, EDARC2, EDARSTC0, EDARSTC1 and EDARSTC2. In addition to the biological samples, one positive control (CM, mock community) and one negative control (NC) were included during PCR amplification to ensure quality control. The mock community was processed in the same manner as the experimental samples. These controls were used exclusively to verify sequencing accuracy and the absence of contamination and were not included in the comparative ecological analyses. The sequencing libraries were prepared and sequenced on an Illumina MiSeq platform (2 × 300 bp). Bioinformatic analysis was conducted by Microomics^®^ S.L. (Barcelona, Spain) following a standard pipeline for metagenomic DNA sequence analysis using QIIME2 and DADA2, as summarised in Figure 1. The raw metagenomic sequences have been deposited in the NCBI BioProject repository under accession number PRJNA 1443304.

#### 2.6.4 Gene prediction models

Functional gene predictions were inferred from the 16S rRNA gene amplicon data using PICRUSt2 (Phylogenetic Investigation of Communities by Reconstruction of Unobserved States, version 2). This tool predicts metagenomic functional profiles in silico, by mapping 16S rRNA sequences to reference genomes and inferring the potential gene content based on phylogenetic relationships. The analysis was carried out in Python 3 using the standard PICRUSt2 pipeline to estimate the functional abundance of KEGG Orthologs (KOs) in each sample. The resulting functional profiles were normalized using the ARiSTa (Adaptive Rank-based Inverse Score Transformation analysis) method prior to statistical testing. Subsequently, Kruskal–Wallis tests were applied to identify significantly different predicted functions among experimental groups. A Random Forest classifier was also implemented to determine the most discriminant predicted functions between treatments. These results should be interpreted as functional predictions derived from 16S data, not as direct metagenomic measurements.

### 2.7 Data processing and statistical analysis

For the study of functional diversity (BiologECO plates^®^), firstly, the kinetics of the samples subjected to different treatments (W irrigation, EDAR and EDAR_ST) and in the presence of PGPB inoculums (C1: *B. pretiosus* CECT30674^T^ and C2: *P. agronomica* CECT30673^T^) were graphed. In order to determine the incubation point of maximum metabolic activity of the community (maximum AWCD value), the slope of each kinetic curve was calculated, and the time point corresponding to the maximum slope was selected as the moment of peak metabolic activity. For that time, metabolic diversity was calculated using the Shannon–Weaver index, the ANOVA mean comparison test was used. When statistically significant differences were detected for a dependent variable (*p*-value < 0.05), a post-hoc pairwise comparison test (Duncan) was performed in order to study which fertigation treatment justified such differences. Finally, to study the effect of the different treatments on the antibiotic resistance profile of the microbial community (cenoantibiogram), a multivariate analysis of principal component analysis (PCA) was used as an exploratory technique of trends and to discriminate differences between treatments. For all analyses, the statistical program SPSS v.29.0 (IBM Corp, Armonk, NY, USA) was used.

## 3 Results

### 3.1 Dry biomass analysis and biometrics of seedlings

**Figure 2:**
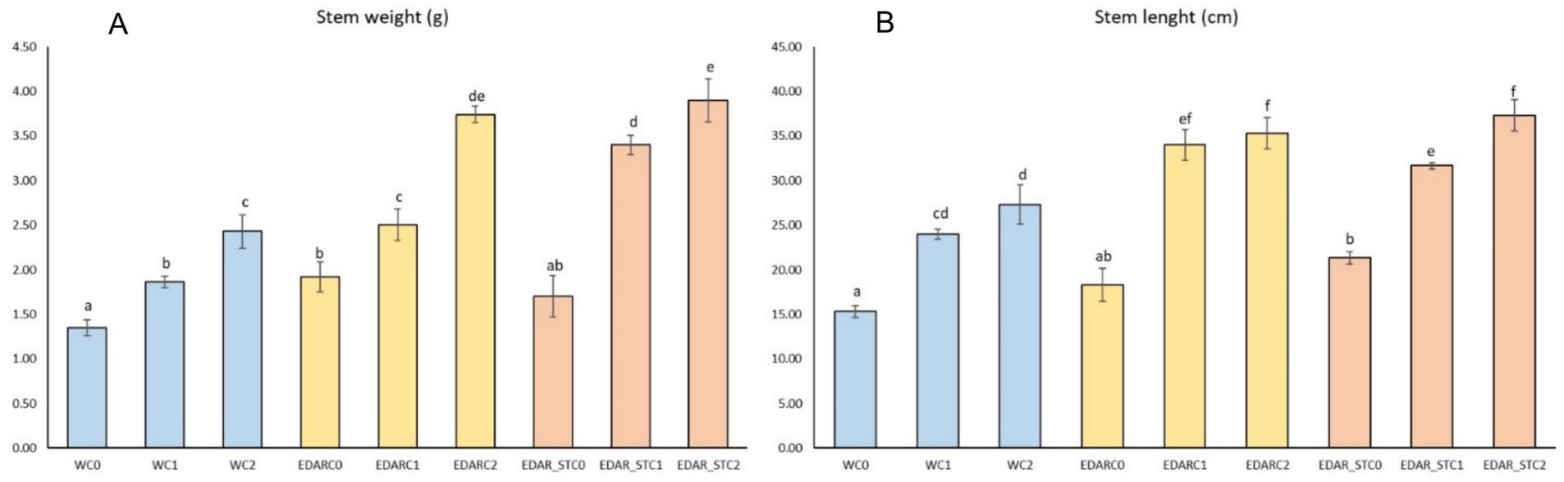
Biometric analysis of **(A)** dry weight of the stem (g) and **(B)** length of the stem (CM). Representation of mean values (*n* = 3). Bars with identical letters indicate that the average values are not significantly different (*p*-value < 0.05). C1 (*B. pretiosus* CECT30674^T^), C2 (*P. agronomica* CECT30673^T^), W (irrigation with water), WWTP (organic fertiliser, named as EDAR), and EDAR_ST (irrigation with sterilized biofertilizer).

As shown in Figure 2, and carrying out an intra-treatment comparison, the incorporation of biological treatments (C1 and C2) improves stem weight significantly. Figure 2 shows the incorporation of C2 in any of the matrices improves significantly the biometric variables measured respect to the control without inoculum (C0) and with respect to the effect of C1. Inter-treatment comparisons revealed that strain inoculation consistently promoted plant growth relative to non-inoculated controls. The incorporation of any of the strains improves the effect of chemical treatment (EDAR and EDAR_ST) on water.

### 3.2 Nutritional composition analysis

**Figure 3:**
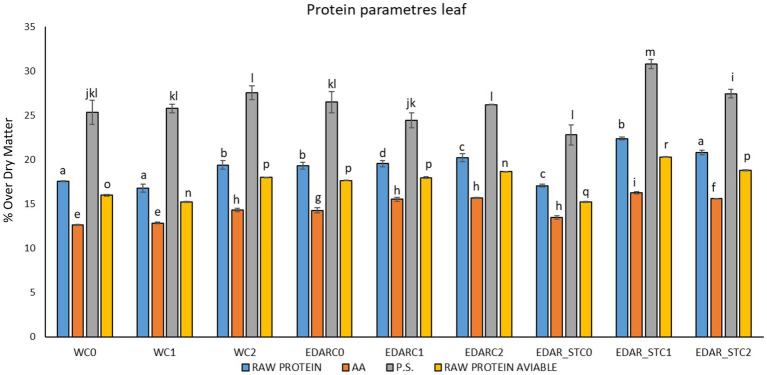
*Quercus suber* L. Mean nutritional variables (*n* = 35) related to protein quality. Bars with identical letters indicate that the average values are not significantly different (*p*-value < 0.05).

**Figure 4:**
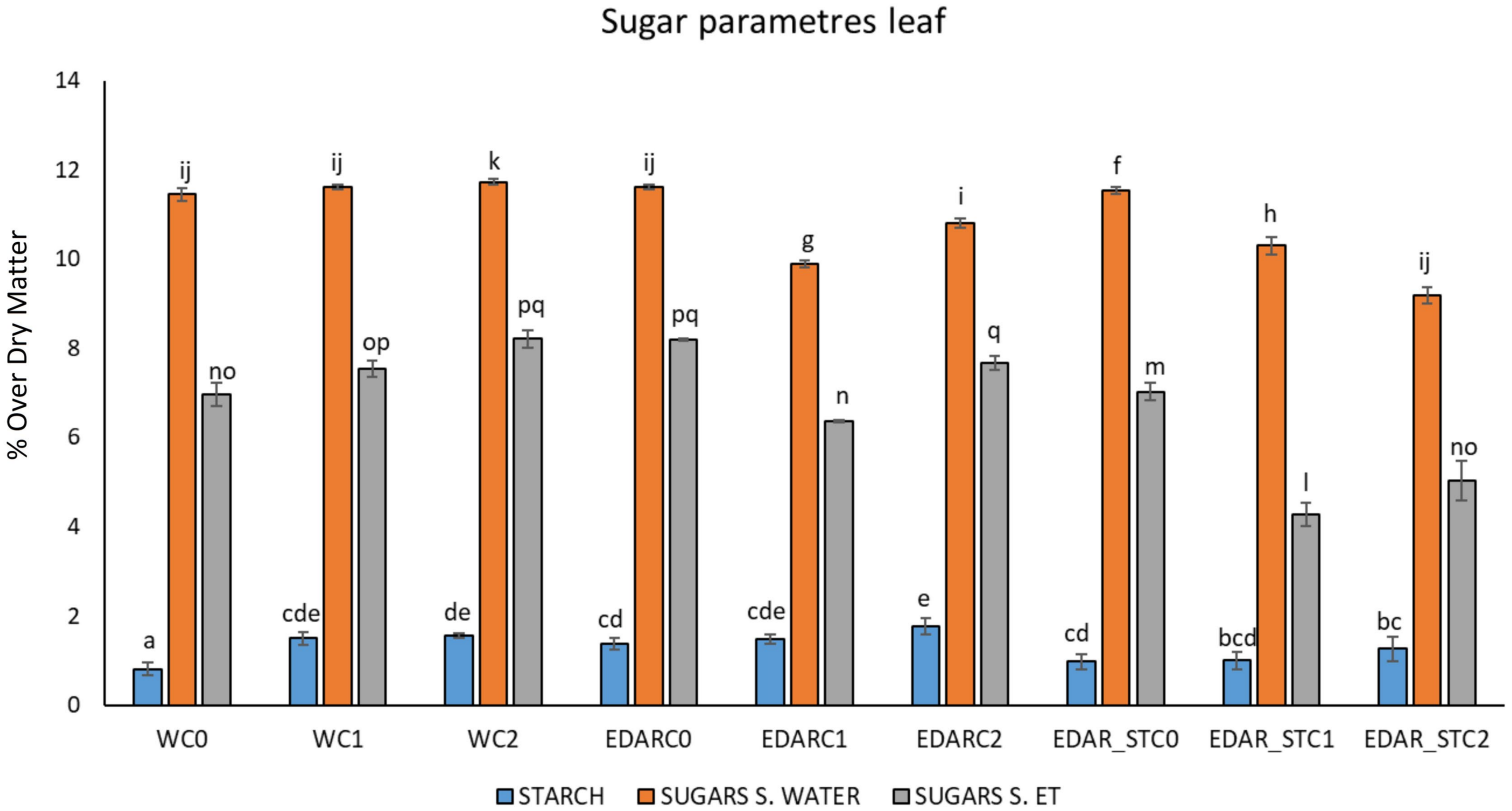
*Quercus Suber* L. Average nutritional variables (*n* = 35) related to sugar quality. Bars with identical letters indicate that the mean values are not significantly different (*p*-value <0.05).

**Figure 5:**
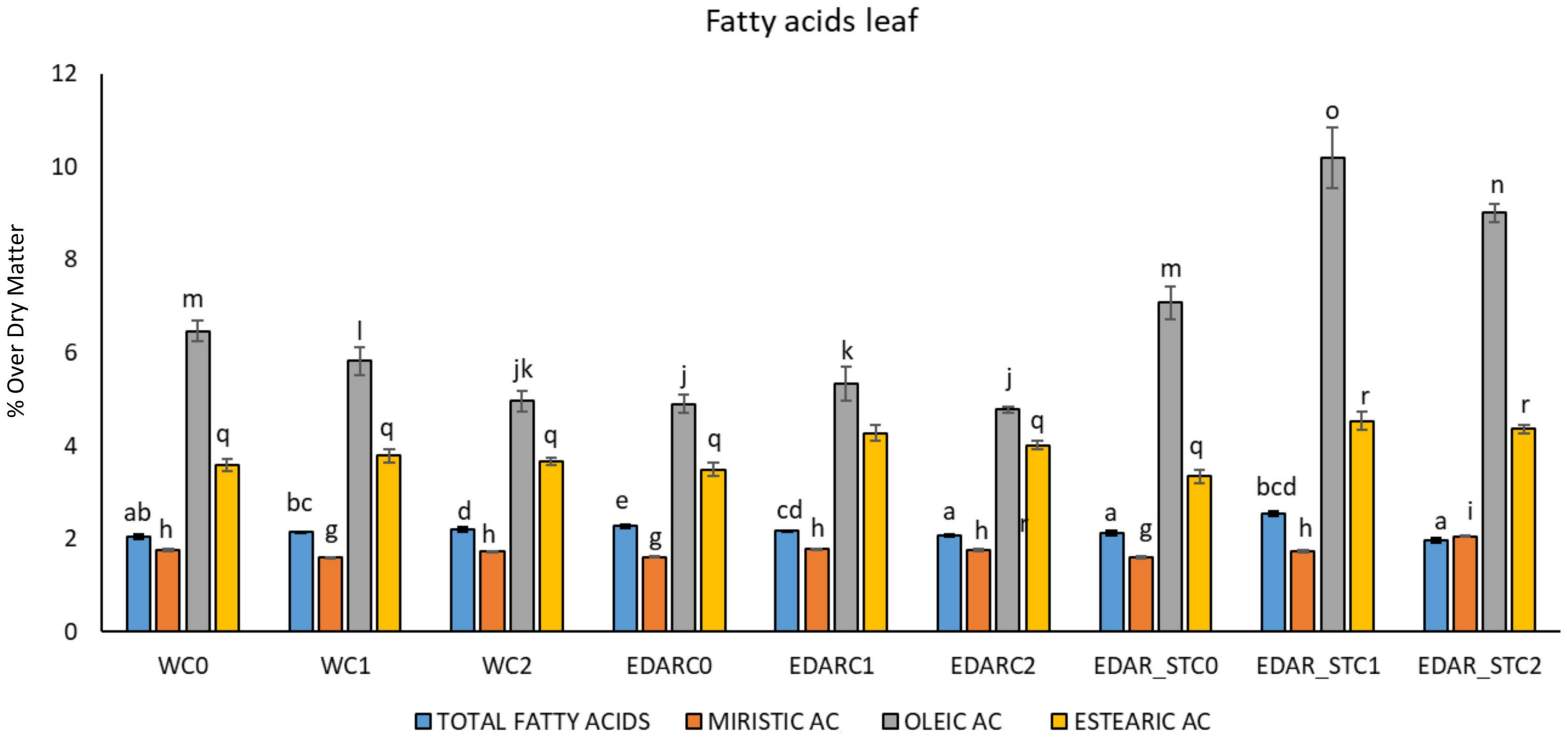
Quercus leaf statistics *Suber* L. Mean nutritional variables (*n* = 35) related to fatty acid quality. Bars with identical letters indicate that the mean values are not significantly different (*p*-value <0.05).

**Figure 6:**
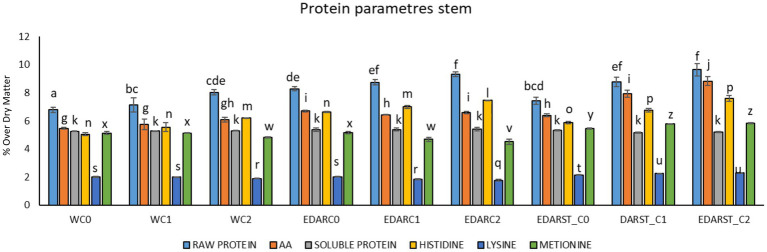
Stem (wood) *Quercus suber* L. Mean nutritional variables (*n* = 35) related to fatty acid quality. Bars with identical letters indicate that the mean values are not significantly different (*p*-value <0.05).

All evaluated variables exhibited statistically significant differences among treatments (*n* = 35 per treatment; *p* < 0.05) according to ANOVA at both the leaf and stem (wood) levels. To further characterize treatment effects, variables for which *post-hoc* analysis (Duncan’s test) indicated significant differences attributable to PGPB inoculation were selected for detailed examination (Figures 3–6). The complete set of graphical representations is provided in the Supplementary material.

Figure 3 presents the leaf protein-related parameters: crude protein (CP), total amino acids (AA), soluble protein (PS), and available protein (PB DISP). In intra-treatment comparisons, inoculation with strains C1 and C2 consistently resulted in significantly higher crude protein (CP) and available protein (PB DISP) levels across all irrigation matrices. In contrast, a significant increase in soluble protein (PS) was observed only under the EDAR_ST irrigation treatment. Differences in total amino acid content were less pronounced; although C1 and C2 inoculation tended to enhance the amino acid profile, these increases were not consistently statistically significant. In inter-treatment comparisons, both raw and sterilized organic fertilization treatments were associated with higher protein-related values relative to water irrigation.

Figure 4 presents the leaf sugar-related parameters: starch, water-soluble sugars (SUGARS S. WATER), and ethanol-soluble sugars (SUGARS S. ET). Both intra- and inter-treatment comparisons revealed comparable patterns. Water-soluble sugars decreased in treatments inoculated with strains C1 and C2 relative to their respective C0 controls within the same irrigation matrix. A similar trend was observed for ethanol-soluble sugars under organic fertilization treatments. In contrast, starch content exhibited a different response pattern. Although differences were less pronounced, inoculation with C1 and C2 resulted in significantly higher starch levels across all irrigation matrices.

Figure 5 presents the leaf fatty acid parameters: total fatty acids, myristic acid (AC. MIRISTIC), palmitic acid (AC. PALMITIC), and stearic acid (AC. STEARIC). In intra-treatment comparisons, palmitic acid content increased significantly across all chemical matrices following inoculation with strains C1 and C2. A similar, although less pronounced, increase was observed for stearic acid. In inter-treatment analyses, both raw and sterilized organic matrices were associated with higher fatty acid levels compared with conventional irrigation using water.

Figure 6 presents the protein-related parameters measured in stem (wood) tissue: crude protein (CP), total amino acids (AA), soluble protein (PS), histidine, lysine, and methionine. In intra-treatment comparisons, soluble protein content was consistently higher in all inoculated treatments relative to their respective non-inoculated controls. Among these, biological treatments combined with sterilized WWTP (EDAR_ST) exhibited the greatest increases compared with their corresponding chemical controls. Across treatments, inoculation with strain C2 resulted in higher soluble protein levels relative to the respective C0 controls. Additionally, WWTP-based treatments showed higher values than water irrigation. Under EDAR_ST conditions, lysine content was also higher in both inoculated treatments (EDAR_STC1 and EDAR_STC2) compared with the non-inoculated EDAR_ST control (EDAR_STC0).

**Figure 7:**
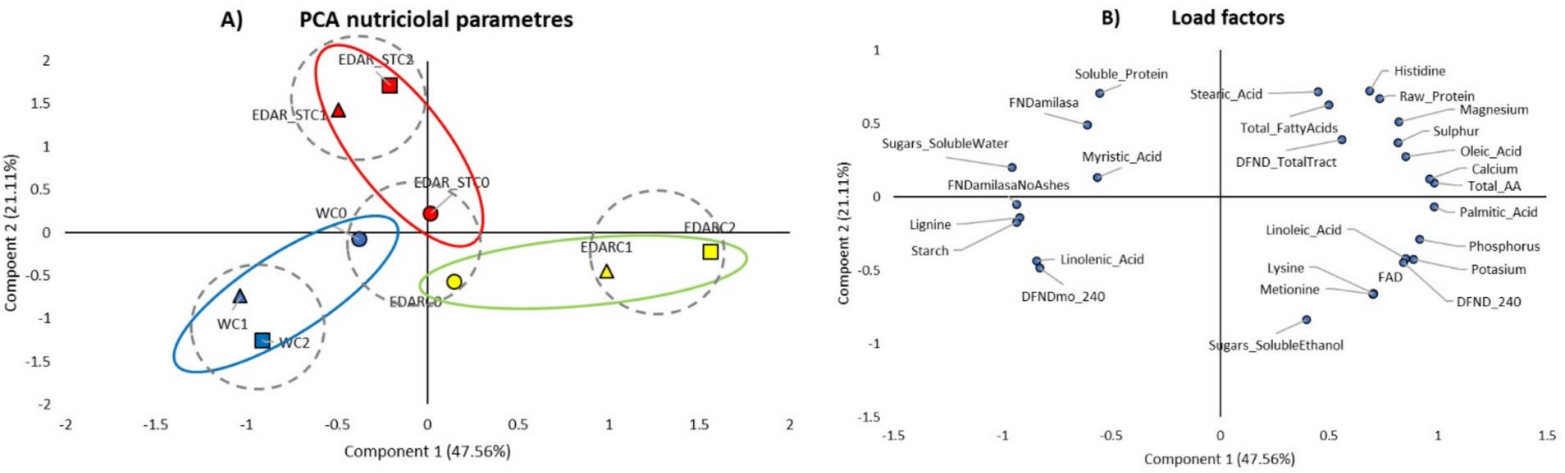
Principal component analysis (PCA) of the nutritional variables measured in *Quercus suber* seedlings under different chemical and biological treatments. **(A)** Two-dimensional ordination plot based on the first two principal components (68.67% cumulative variance explained). Samples are coloured according to chemical treatment: water irrigation (W, blue), WWTP biofertilizer (green—organic fertiliser, named as EDAR), and sterilized WWTP (EDAR_ST, red). **(B)** Loading plot showing the contribution of nutritional variables to the principal components.

To obtain an integrative representation of the nutritional dataset, principal component analysis (PCA) was performed. The resulting two-dimensional projection enabled visualization of clustering patterns associated with the different fertigation treatments. As shown in Figure 7A, the first two principal components accounted for 68.67% of the total variance. Non-inoculated plants (C0), irrespective of the chemical matrix, were distributed predominantly in the central region of the ordination space. In contrast, inoculated treatments exhibited a clear separation along the principal axes, indicating that biological treatment was a major driver of multivariate differentiation. Samples treated with strains C1 and C2 were positioned toward the extremes of the ordination plot, suggesting distinct nutritional profiles associated with each inoculant. A further level of segregation was observed according to the chemical matrix: WWTP treatments (green) clustered in the lower right quadrant, EDAR_STC2 (red) in the upper left quadrant, and water-treated samples (blue) in the lower left quadrant. The loading plot (Figure 7B) indicates that variables contributing positively to PC1 are primarily associated with WWTP and EDAR_ST treatments, whereas variables with negative loadings are predominantly linked to water-treated samples. These patterns suggest that both the biological inoculum and the chemical matrix jointly shaped the multivariate nutritional profile of the seedlings.

### 3.3 Analysis of functional diversity

Metabolic diversity was quantified for each sample using the Shannon–Weaver diversity index. Statistical comparisons were performed at 144 h of incubation, corresponding to the stationary phase of microbial growth. Samples were classified according to chemical treatment: W (water irrigation), WWTP (organic fertiliser, named as EDAR), and EDAR_ST (sterilized biofertilizer); and biological treatment: C0 (non-inoculated control), C1 (*Bacillus pretiosus* CECT30674^T^), and C2 (*Pseudomonas agronomica* CECT30673^T^). No statistically significant differences in functional diversity were detected among chemical or biological treatments (Table 3). Shannon index values were comparable across treatments, indicating stable functional diversity and evenness. Furthermore, inoculation with strains C1 or C2 did not significantly alter functional diversity within any of the evaluated chemical matrices.

**Table 3:** Functional diversity (Shannon index, H) of the soil microbial community.

	WC0	WC1	WC2	EDARC0	EDARC1	EDARC2	EDARSTC0	EDARSTC1	EDARSTC2
H′ 120 h	4.61 ± 0.02	4.73 ± 0.88	4.62 ± 1.12	4.44 ± 1.39	4.78 ± 0.79	4.39 ± 1.78	4.84 ± 2.04	4.16 ± 1.17	4.87 ± 0.88

### 3.4 Analysis of community antibiotic resistance: cenoantibiogram

**Figure 8:**
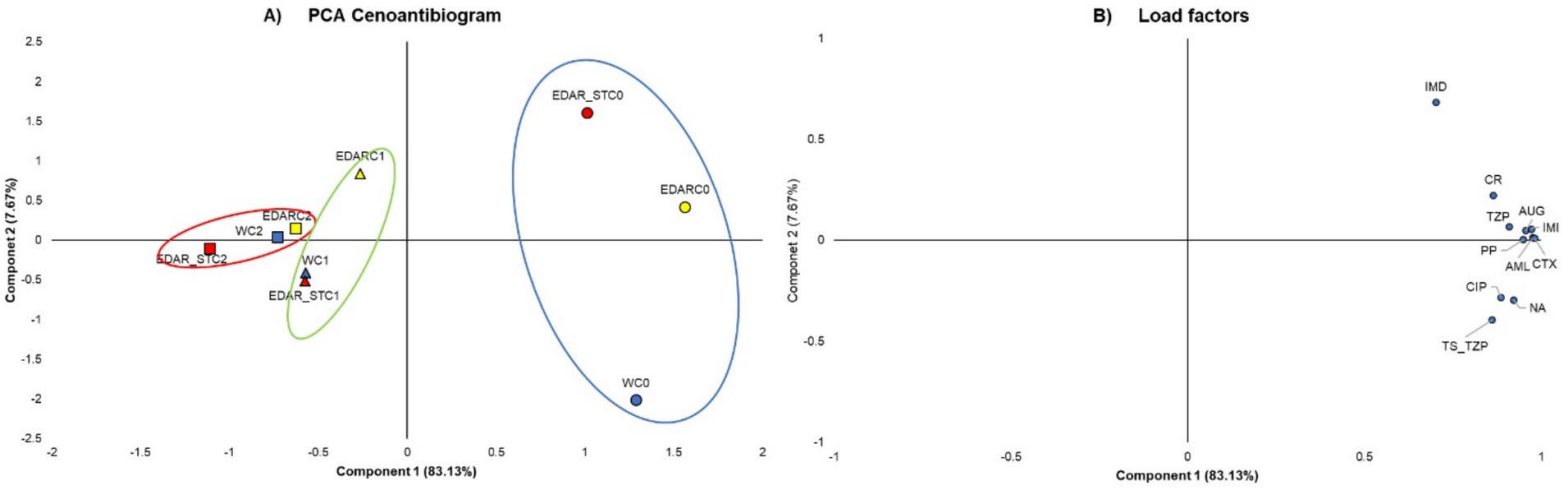
**(A)** PCA that represents in the (2D) plane the distribution and variation trends of chemical and biological irrigation treatments, according to the two components (variables: antibiotics), which best explain the model. C0: control without inoculum. C1: *B. pretiosus* CECT30674^T^. C2: *P. agronomica* CECT30673^T^. W: watering with water. Surrounded in blue, a grouping of treatments irrigated with the Controls (C0). Surrounded in green, grouping of treatments irrigated with *B. pretiosus* CECT30674^T^ (C1). Surrounded in red, a grouping of treatments irrigated with *P. agronomica* CECT30673^T^ (C2) and **(B)** Load factors.

Figure 8A presents the principal component analysis (PCA) based on the two components that account for 90.8% of the cumulative variance. The ordination plot reveals a clear separation of samples according to biological treatment, reflecting distinct antibiotic resistance profiles among groups. Non-inoculated plants (C0), irrespective of the chemical matrix, cluster predominantly on the right side of the plot, whereas treatments inoculated with strains C1 and C2 are distributed toward the left. A further level of differentiation is observed between biological treatments, with C1 (green) and C2 (red) occupying distinct regions of the ordination space. The loading plot (Figure 8B) indicates that most tested antibiotics are associated with the right-hand side of the PCA, suggesting that these variables contribute most strongly to the separation of non-inoculated treatments (C0) toward higher resistance values.

### 3.5 Metagenomic study of the rhizosphere

In the metagenomic analysis, a total of 648,953 valid reads were obtained from the sequencing of the 16S rRNA amplicon after clipping (BioProject PRJNA1443304). The mean amplicon length was 301 bp.

A total of nine treatments were analysed, each in triplicate, resulting in 27 soil samples subjected to metagenomic sequencing. The number of valid sequences detected for each soil sample exceeded 14,171. The alpha rarefaction curves reached a plateau in all samples, indicating that sequencing depth was sufficient to provide a reliable estimation of total taxonomic diversity.

The metagenomic results presented in this manuscript correspond to the comparative analysis among the nine treatments, including the control treatment. The control samples were used as a baseline to assess changes in microbial community composition and diversity across treatments.

#### 3.5.1 Beta diversity

Beta diversity was assessed using Principal Coordinates Analysis (PCoA) based on the Weighted UniFrac distance metric, which considers both phylogenetic relationships and relative abundance among taxa.

**Figure 9:**
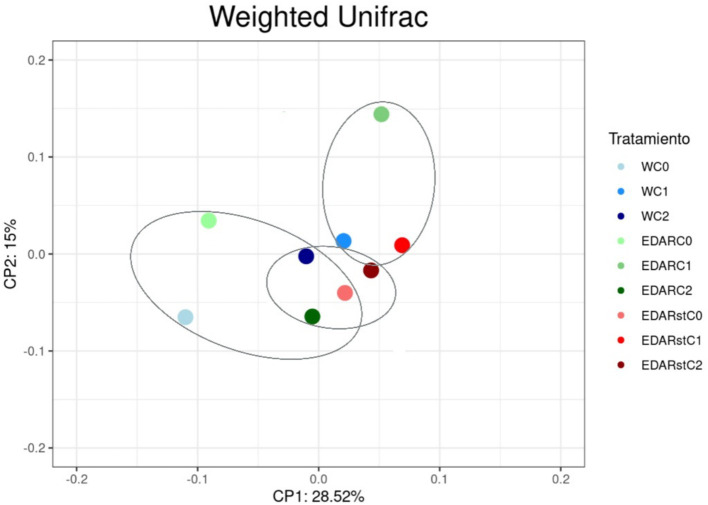
ACP that represents in the plane (2D) the distribution and trends of variation of the taxonomic diversity (Weighted Unifrac) of the samples, depending on the chemical treatment: W (irrigation with water), WWTP (organic fertiliser, named as EDAR) and EDAR_ST (with sterile biofertilizer) and biological C0 (control without inoculum), C1 (*B. pretiosus* CECT30674^T^), C2 (*P. agronomica* CECT30673^T^), which best explain the model. The chemical treatments watered with the residue are coloured in greenish tones, the grouping of the chemical treatments watered with the sterilised residue is coloured in reddish tones. Coloured in bluish tones, grouping of chemical treatments irrigated with water (W). Surrounded in gray, according to grouping trends by biological treatment.

As shown in Figure 9, the distribution of samples does not reveal a clear segregation by treatment, although some grouping tendencies can be observed within the inoculated communities (C1 and C2) compared to their respective controls (C0). The first two coordinate axes explained 28.52% (PC1) and 15.19% (PC2) of the total variation in community composition.

The PERMANOVA test (Table 4) confirmed that treatment had no statistically significant effect on the overall bacterial community structure (pseudo-*F* = 3.65, *R*^2^ = 0.619, *p* = 1). Similarly, the ANOSIM test (Table 5) showed a moderate degree of separation among treatments (*R* = 0.736) but without statistical significance (*p* = 1). The PERMDISP analysis (Table 6) indicated no significant differences in dispersion among groups (*p* = 0.564), suggesting that the observed patterns are not attributable to heterogeneity of variances.

**Table 4:** Summary of statistical analyses associated with beta diversity (Weighted UniFrac).

PERMANOVA	Df	SumsOfSqs	MeanSqs	F. Model	*R^2^*	*p*-value
Treatment	8	0.37374	0.04672	365.284	0.61883	1
Residuals	18	0.23021	0.01279		0.38117	
Total	26	0.60394			1	

The table shows the output of three tests: PERMANOVA (permutational multivariate analysis of variance). For PERMANOVA, the table includes the degrees of freedom (Df), sum of squares (SumsOfSqs), mean squares (MeanSqs), pseudo-*F* value (F. Model), coefficient of determination (*R*^2^), and *p*-value.

**Table 5:** ANOSIM (analysis of similarities) and PERMDISP (permutational analysis of multivariate dispersion).

ANOSIM	ANOSIM statistic *R*	Significance
Treatment	0.73594	1

The ANOSIM section reports the *R* statistic and its corresponding significance level.

**Table 6:** The PERMDISP section lists the degrees of freedom, sum of squares, mean squares, *F*-value, and *p*-value, summarizing the homogeneity of multivariate dispersion among groups.

PERMDISP	Df	Sum Sq	Mean Sq	*F*-value	Pr(>F)
Groups	8	0.00364	0.00045	0.86181	0.56441
Residuals	18	0.0095	0.00053		

Altogether, these results indicate that, according to the Weighted UniFrac metric, the inoculation treatments did not produce statistically significant changes in the phylogenetic structure of the rhizospheric bacterial communities, although some visual trends in community clustering can be observed.

#### 3.5.2 Relative abundances at the genus level

**Figure 10:**
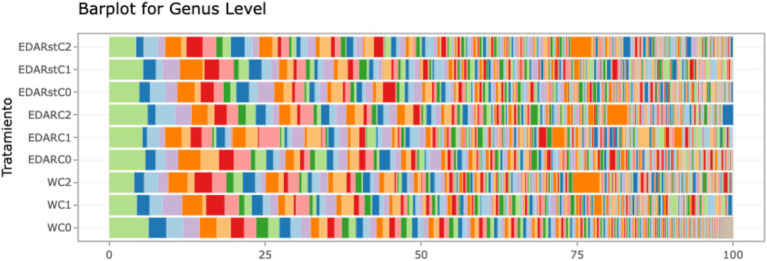
Relative abundances of taxonomic composition at the genus level of rhizospheric samples of *Q. suber* under the different chemical and biological treatments. C0: control without inoculum. C1: *B. pretiosus* CECT30674^T^. C2: *P. agronomica* CECT30673^T^. W: watering with water.

To complement the diversity analysis, the relative abundances of bacterial genera in the rhizospheric samples of *Q. suber* under different chemical and biological treatments were evaluated (Figure 10).

In the treatments inoculated with strain C1 (*B. pretiosus* CECT30674^T^) or strain C2 (*P. agronomica* CECT30673^T^), an increase in the relative abundance of the corresponding genera (*Bacillus* and *Pseudomonas*, respectively) was observed. This pattern suggests that the introduced plant growth-promoting bacteria (PGPB) may have persisted and integrated into the native microbial community over time, without displacing pre-existing taxa.

Figure 10 highlights the genera that coincide with the bacterial inoculants incorporated in the treatments, illustrating their relative increase in the corresponding inoculated samples. The complete taxonomic assignments at the genus level are provided in Supplementary Table S2.

#### 3.5.3 Gene prediction

**Figure 11:**
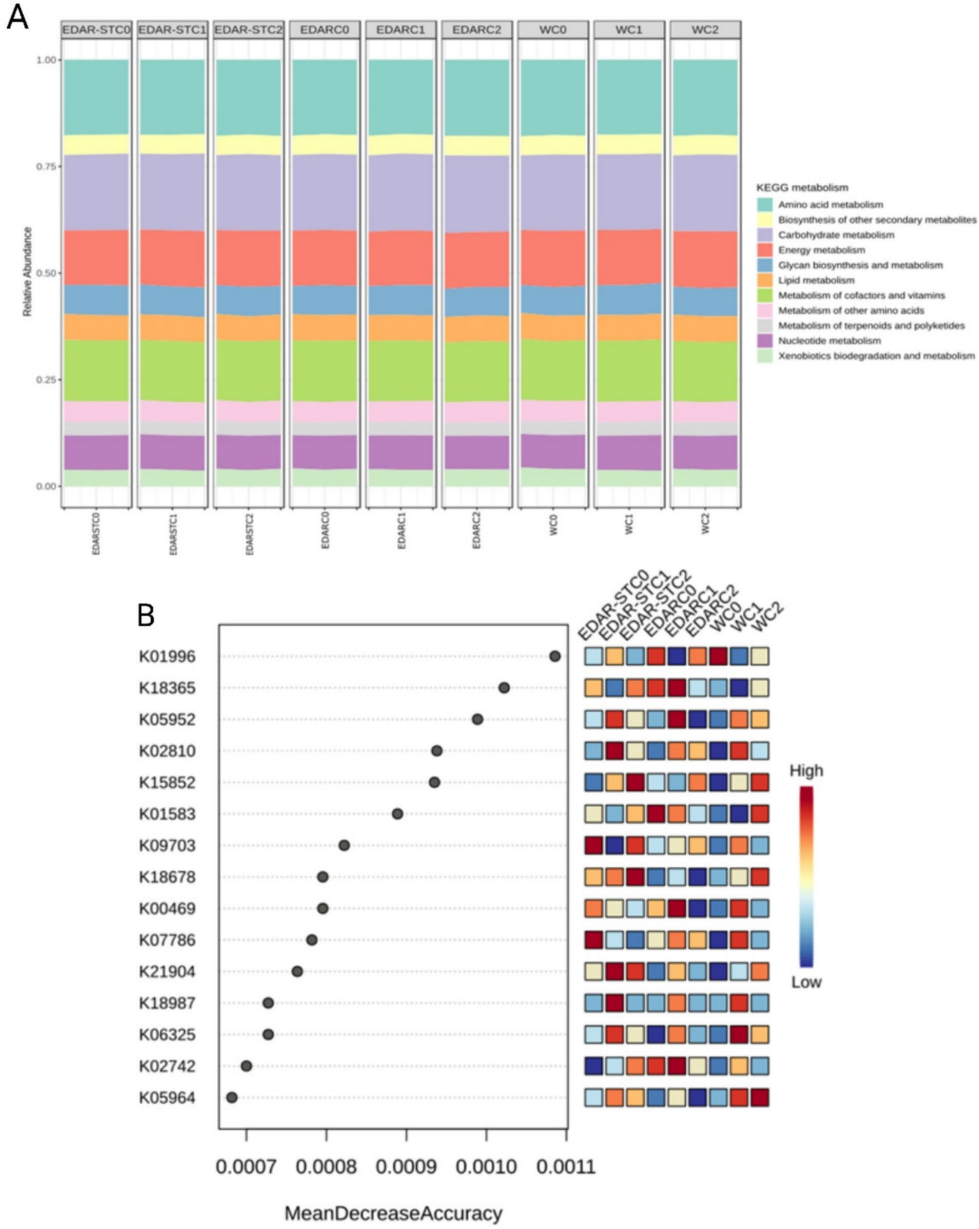
**(A)** General functional profile of KEGG metabolic pathways predicted by PICRUSt2 in microbiomes associated with water and biofertilizer treatments (sterilized and non-sterilized). The main routes correspond to level 2 categories of the KEGG system. **(B)** KEGG functions of greatest importance in the Random Forest classification model for discrimination between treatments. The functions represented correspond to the KOs with the highest “Mean Decrease Accuracy” in the model.

A functional analysis based on predicted KEGG annotations inferred from 16S rRNA data was carried out to characterize the predicted metabolic potential of the microbial communities present in the different treatments: WWTP biofertilizer, its sterilized version EDAR_ST and water treatment. Functional profiles were grouped into major metabolic pathways and visualized using relative abundance plots (Figure 11A).

Overall, the microbiomes of all treatments showed a similar predicted functional distribution, dominated by pathways predicted to be associated with amino acid metabolism, carbohydrate metabolism, cofactor and vitamin metabolism, and energy metabolism. Pathways related to the biosynthesis of secondary metabolites, lipid and nucleotide metabolism, and xenobiotic degradation were also predicted to a lesser extent.

A differential analysis of predicted KEGG functional pathways was performed among microbial communities using the nonparametric Kruskal–Wallis test. No predicted function reached significance after correction for multiple tests (FDR > 0.05).

A classification analysis was applied using Random Forest based on KO functions predicted by PICRUSt2, with the aim of identifying the most relevant predicted functional categories for discrimination between treatments (Figure 11B).

The results show that the model reached an overall classification error (OOB error) of 55.6% and allowed the identification of a subset of predicted KO functions with a high contribution to the accuracy of the model, measured as “Mean Decrease Accuracy.”

The Random Forest-based classification using PICRUSt2-predicted functions (i.e., inferred functional potential rather than directly measured metagenomic functions) allowed the identification of putative discriminant functional trends between treatments by combining different matrices and bacterial inoculants (Table 7). In the water controls, WC0 showed a higher predicted potential for genes putatively related to active amino acid transport (K01996), while WC2 showed a predicted enrichment of pathways potentially involved in lipid biosynthesis pathways by holo-ACP synthase (K05964). In contrast, WC1 was predicted to exhibit an increased functional potential for genes encoding a spore coat protein (K06325), possibly related to specific bacterial structures.

**Table 7:** Discriminant KEGG functions between treatments identified by classification with random forest.

Treatment	KO	Predicted function (KEGG)
WC0	K01996	Branched-chain amino acid transport system ATP-binding protein
WC1	K06325	Spore coat protein B
WC2	K05964	Holo-ACP synthase [EC:2.7.7.61]
EDARC0	K01583	Arginine decarboxylase [EC:4.1.1.19]
EDARC1	K18365	4-hydroxy-2-oxovalerate/4-hydroxy-2-oxohexanoate aldolase [EC:4.1.3.39, 4.1.3.43]
	K00469	Inositol oxygenase [EC:1.13.99.1]
	K02742	Protein SprT (uncharacterized)
EDARC2	—	No outstanding differential features
EDARSTC0	K09703	Uncharacterized protein
	K07786	MFS transporter, DHA2 family, multidrug resistance protein
EDARSTC1	K02810	Sucrose PTS system EIIBCA/EIIBC component [EC:2.7.1.211]
	K21904	Metallothionein
	K18987	Two-component system, OmpR family, response regulator Irr
EDARSTC2	K15852	LuxR family transcriptional regulator
	K18678	Phytol kinase [EC:2.7.1.182]

The treatments applied, the KEGG orthologs identifiers (KO) predicted by PICRUSt2, their associated functions and the possible functional interpretation are shown.

The EDARC1 treatment was predicted to be enriched in functions putatively associated with the catabolism of aromatic compounds (K18365), oxidation of inositols (K00469), and protein processing (K02742), suggesting a predicted activation of metabolic capabilities rather than confirmed activity. In EDARC0, a higher predicted abundance of genes encoding arginine decarboxylase (K01583), involved in the production of polyamines, was inferred. No notable predicted differential functions were identified in EDARC2.

Treatments with sterilized WWTPs also showed differences in predicted functional potential in relation to inoculation. EDARSTC1 was predicted to be enriched in functions putatively related to sugar uptake (K02810), response to metals (K21904), and regulation of environmental stress (K18987), while EDARSTC2 showed a predicted enrichment of functions putatively related to transcriptional regulation such as quorum sensing (K15852) and phytol phosphorylation (K18678). On the other hand, EDARSTC0 (without bacteria) was characterized by predicted functions putatively associated with resistance to toxic compounds (K07786) and uncharacterized proteins (K09703), suggesting baseline predicted functional potential after sterilization.

## 4 Discussion

Ecosystem stability and functional integrity are closely associated with microbial diversity, as soil microorganisms play fundamental roles in resistance and resilience to environmental disturbances, including climate change and wildfires (Allison and Martiny, 2008; Santillan et al., 2025). Wildfire events have been shown to substantially alter soil microbial communities, often reducing total microbial biomass in the short term while favoring opportunistic taxa that rapidly proliferate under altered nutrient regimes (Allison and Martiny, 2008; Santillan et al., 2025). Soils characterized by higher microbial diversity and structural stability generally exhibit greater recovery potential, thereby facilitating vegetation regeneration and the restoration of ecosystem functions (Li et al., 2024b).

In this context, the selection of *Bacillus pretiosus* CECT30674^T^ (C1) and *Pseudomonas agronomica* CECT30673^T^ (C2) was based on their previously demonstrated plant growth-promoting (PGPB) capabilities and biotechnological potential (Robas Mora et al., 2023). These strains have demonstrated their ability to promote growth, not only in herbaceous plants (Mora et al., 2022; Robas Mora et al., 2023; Fernández Pastrana et al., 2025) but also in other models of Fagaceae (Fernández-Pastrana et al., 2025a,b; Penalba-Iglesias et al., 2025). These strains have been characterized at the genomic level and shown to lack virulence determinants and transmissible antibiotic resistance genes, supporting their environmental biosafety upon inoculation (Robas Mora et al., 2023). Although originally isolated from the rhizosphere of *Medicago sativa*, their occurrence in natural systems suggests that their ecological functionality may extend beyond agricultural contexts, potentially contributing to biodiversity conservation and ecosystem stability in forest environments.

In the present study, WWTP sludge fulfilled a dual function: serving both as a carrier matrix for PGPB inoculants and as a complex nutrient source. Through microbial activity, organic constituents within the sludge can be mineralized into inorganic forms, thereby enhancing nutrient availability for plant uptake (Ferreira et al., 2023). Comparable valorisation strategies have been explored in the recovery of municipal solid waste, agricultural residues, and microalgal biomass (Devi and Sumathy, 2017; Ferreira et al., 2025). Similar approaches have also been implemented in reforestation programs involving pine species (Nazarov et al., 2023; Garcia-Villaraco et al., 2024).

A growing body of evidence indicates that specific bacterial strains can play a pivotal role in ecosystem restoration by promoting plant growth and accelerating biomass accumulation (Peddle et al., 2025; Singh and Srivastava, 2025). Consistent with these findings, inoculation with *Bacillus pretiosus* CECT30674^T^ and *Pseudomonas agronomica* CECT30673^T^ enhanced seedling biomass and stem length across chemical matrices relative to their non-inoculated controls. Moreover, seedlings receiving WWTP-based biofertilizer (raw or sterilized) combined with C1 or C2 exhibited greater biometric development than those subjected to conventional water irrigation, either with or without inoculation. Similar growth-promoting effects of Bacillus spp. have been reported in pine systems, where inoculation resulted in increased biomass production and root elongation (Puri et al., 2020).

The use of WWTP-derived matrices is particularly relevant due to their complex nutrient composition, which can be transformed by PGPB into plant-assimilable inorganic forms, thereby enhancing nutrient bioavailability (Asghar et al., 2020). Notably, under EDAR_ST conditions, a significant increase in stem length was observed compared with other chemical treatments. This effect may be associated with the removal of indigenous microbial competitors during sterilization, potentially allowing more efficient resource transformation by the introduced strains (Shen and Zhang, 2015; Saif et al., 2021). Specifically, the results obtained demonstrate how the C2 strain (*P. agronomica* CECT30673^T^) stimulated plant growth both in EDAR and in EDAR_ST, having a greater cost–benefit potential in its subsequent use as a biofertilizer. A similar effect was reported by Tang et al. (2022), who used WWTP effluent as a matrix for crop plants following alkaline thermal hydrolysis pretreatment to enhance its fertilization potential. Sludge sterilization (WWTP) ensures the competitiveness of *Bacillus* and *Pseudomonas* strains by eliminating the native microbiota and pathogens (Herrmann and Lesueur, 2013), assuring scientific reproducibility and biosafety. However, this process significantly increases operating expenditure (OpEx) and carbon footprint and can alter the chemical integrity of the substrate and volatilize essential nutrients (Mahdi et al., 2010; O’Callaghan, 2016). Therefore, although it is vital for experimental rigor, its industrial scalability requires evaluating less expensive stabilization alternatives that maintain the viability of the biofertilizer.

Furthermore, knowing the nutritional status of plants can be used as an indirect measure of their adaptive capacity and resilience (Karlidag et al., 2007; Flajšman et al., 2019; Bourdon et al., 2021). The present study shows that those plants inoculated with the C1 (*B. pretiosus* CECT30674^T^) and C2 (*P. agronomica* CECT30673^T^) strains significantly improved the nutritional status of the plant model in several variables, mainly those related to stem robustness, which potentially translates into greater strength in response to abiotic stress and potential long-term survival (Kumari et al., 2022).

In various investigations on the inoculation of plant growth-promoting bacteria, significant differences have been observed related to variability in efficacy according to soil type, environmental conditions, and competition with native microorganisms. Works such as that of Wang N. R. et al. (2022) found that the efficacy of *Pseudomonas fluorescens* varied with different soil types. Similarly, it is known that positive results obtained under controlled laboratory conditions were not always replicated under field conditions (Lucy et al., 2004; Schmidt and Gaudin, 2018). This underscores the translational value of controlled studies such as the present one as a prerequisite for field-scale application.

The potential impact of exogenous bacterial inoculants on native rhizospheric communities also warrants investigation (da Cunha et al., 2024). It is known that, in general, the introduction of non-native and exotic species in an ecosystem affects its biological diversity (Wang et al., 2020). Although there are some exceptions to this postulate in the field of microbiological ecology. The introduction of a new species can lead to an increase in diversity without the need to displace the rest of the soil community (Wang et al., 2020).

In microbial ecology, the Shannon index of Shannon (2001) and Konopiński (2020) is commonly used to assess community diversity. In the present study, this index has been used for the metabolic and functional diversity of the communities of the soils under study. The results indicate that metabolic diversity remained stable regardless of the applied treatment. This may be due to the shielding effect of the soil, which is very rich in nutrients. Therefore, the stability of edaphic functional diversity can be interpreted as an expected biological behaviour (Rosas et al., 2022). Rhizospheric communities seem to be able to host exogenous strains that promote different positive effects on the plant, without altering the functionality of the community (Ambrosini et al., 2016).

It is known that the addition of a strain to a biological system can alter the antibiotic resistance profile of the soil community that hosts them (Yang et al., 2022; Robas Mora et al., 2023). It is therefore interesting to analyse the impact on the antibiotic resistance profile that the incorporated strains can produce. Once incorporated into the soil, they can displace more resistant phenotypes, reducing the MIC of the soil microbiota, reducing the horizontal transfer of these resistances (Ambrosini et al., 2016; Yang et al., 2022). Previous studies described the phenotypes of the PGPB strains used in the present study, showing that they have low levels of MIC compared to various antibiotics of widespread clinical use in human and animal medicine (Robas Mora et al., 2023). Consistently, in the present work it was observed how the addition of PGPB strains induces a decrease in soil community MICs. The analysis of this fact from a “One Health” perspective allows us to interpret a bioprotective effect by minimizing the horizontal transmission of antibiotic resistance genes. Previous studies highlight this same fact, looking at how the addition of a strain could mitigate the resistance profile of the soil community (González-Reguero et al., 2023; Fernández-Pastrana et al., 2025a,b), yet few studies have examined the capacity of PGPB to reduce soil community MICs upon inoculation. This also provides an opportunity to explore new research directions that offer new evidence on the behaviour of soil communities in terms of antibiotic resistance profiles and how to mitigate its effect and potential transmission in the food chain.

The observed reduction in resistance profiles following inoculation with strains C1 and C2 (Supplementary material) suggests a potential mitigating effect on antibiotic resistance phenotypes (González-Reguero et al., 2023). In inoculated samples, ordination analysis (Figure 8; cumulative variance = 90.8%) revealed clear differentiation according to the presence or absence of inoculum and the specific strain applied, indicating that biological treatment was associated with distinct resistance patterns. The clustering of non-inoculated samples (C0) alongside antibiotic loading factors suggests comparatively higher resistance values under non-inoculated conditions. Collectively, these findings are consistent with the hypothesis that strain inoculation may contribute to a reduction in resistance phenotypes within the soil microbiota. Given that soils represent major environmental reservoirs of antibiotic resistance genes (Forsberg et al., 2012), such modulation of resistance profiles may have broader ecological implications. However, further validation under field conditions would be required to confirm the long-term stability and ecological relevance of these effects.

Over recent decades, a wide range of molecular approaches has been developed to investigate complex microbial communities (Leonard et al., 2020; Mahdi et al., 2022; Zou et al., 2025). These methodologies have enabled increasingly detailed characterization of microbial composition, functional potential, and ecological dynamics (Yang et al., 2025). Among them, metagenomics represents a key framework for evaluating treatment-induced shifts in soil microbiomes (Espinosa-Asuar et al., 2014). Amplicon-based metagenomic analysis targeting the 16S rRNA gene provides high-resolution insight into the taxonomic composition and relative abundance of microbial communities (Babalola et al., 2025; Mishra et al., 2025). In this context, such approaches are instrumental for assessing whether observed plant responses are associated with shifts in rhizospheric microbial structure following PGPB inoculation. Previous studies have shown that fertigation regimes can influence taxonomic diversity, although the magnitude and direction of these effects depend on factors such as community stability, physicochemical soil properties, and granulometric composition (Wang S. et al., 2022).

In the present study, inoculation with *B. pretiosus* CECT30674^T^ and *P. agronomica* CECT30673^T^ increased the relative abundance of the corresponding genera (*Bacillus* and *Pseudomonas*) without significantly altering overall taxonomic diversity. These findings suggest that the introduced strains could persist within the rhizosphere and integrated into the existing microbial community without causing detectable disruption, but more detailed genomic studies in the future are needed to confirm the persistence of the strains. Similar integration patterns have been reported in other systems, where inoculated strains increased in abundance while maintaining native community structure (Armada et al., 2018; Dobrzyński et al., 2025).

Several metagenomic studies have studied the effects of PGPB on plants, focusing on why these bacteria induce changes in plants (Ahmed et al., 2025; Alharbi et al., 2025). Moreover, it is known that the significant differences in treatments with the added strains are due to the fact that the rhizospheric and the endophytic microbial community play equally important roles in the complicated plant-microbe interaction (Hawxhurst et al., 2023). Furthermore, other studies (Mattana et al., 2014; Serwecińska, 2020; Ma et al., 2025) show how the application of WWTP sludge allows the composition of the soil communities in which they are applied to be beneficially modified. In the present study, the addition of the strains *B. pretiosus* CECT30674^T^ and *P. agronomica* CECT30673^T^ to the recovered residue yielded higher values than the residue alone. Moreover, this approach not only promotes plant growth but also represents a promising strategy for sustainable agriculture and forestry, enabling waste valorisation while reducing reliance on chemical fertilizers, by offering an effective and sustainable solution to optimize plant production, promote global food security, and revalue waste. Despite the benefits found, it is essential to continue research to ensure that its application is safe and effective, minimizing the risks associated with the introduction of contaminants and pathogenic microorganisms or antibiotic resistance genes.

Functional prediction revealed a conserved metabolic core in all treatments, dominated by functions essential for microbial survival. However, when comparing the functional profiles, subtle differential patterns emerge attributable to the type of treatment and the interaction with bacterial inoculants.

Water treatments (WC0) served as a reference to observe the behaviour of the native microbiota without additional nutrient input. The activation of amino acid transport functions in WC0 suggests a survival strategy based on the efficient capture of scarce resources. In contrast, WC1 and WC2, with *B. pretiosus* CECT30674^T^ and *P. agronomica* CECT30673^T^ respectively, exhibited structural and biosynthetic traits that imply a modulation of the microbiome by inoculation, although in nutritionally limiting contexts. This is consistent with results obtained where it is observed that the flexibility of the microbiome, through association with beneficial bacteria, can facilitate rapid responses to environmental change, providing an alternative route for the adaptation of organisms (Voolstra and Ziegler, 2020).

In comparison, the unsterilized residue (EDARC0) generated greater functional differentiation, especially in the presence of *B. pretiosus* CECT30674^T^ (EDARC1), where complex catabolism and processing pathways were activated. This could reflect a synergy between residue compounds and the activity of the inoculated bacterial strain, facilitating the expression of functions not observed in water or in the residue without bacteria (EDARC0). The high representation of arginine decarboxylase in EDARC0, absent in other treatments, suggests a response of the native microbiome to the chemical stress of the residue without external competition.

The effect of waste sterilization (EDARST) evidenced the specific role of each strain *B. pretiosus* CECT30674^T^ (EDARSTC1) induced functions related to environmental adaptation (sugar uptake, response to metals), possibly favoured by the absence of competitive microbiota. On the other hand, *P. agronomica* CECT30673^T^ in EDARSTC2 activated transcriptional regulation pathways, such as quorum sensing, associated with functional reorganization in sterile environments. The lack of functional activity highlighted in EDARSTC0 reinforces the hypothesis that sterilization reduces functional complexity, which is only restored by inoculation. This pattern is consistent with findings from other studies showing that interactions between species may limit abiotic adaptation in soil bacterial communities, as the adaptive benefits of adaptive mutations are nullified in more complex communities (Hall et al., 2018).

Comparing the three matrix contexts (water, non-sterile waste and sterilized waste), it is observed that functional complexity depends on both the type of substrate and the interaction with the bacterial inoculum. While water limits functional expansion, the residue, especially unsterilized, acts as an activating matrix. Sterilization, on the other hand, reduces baseline functional diversity, but allows the specific effect of each strain to be observed more clearly.

These results suggest that microbial functions do not emerge uniformly across treatments, but result from specific interactions between matrix, physicochemical state and bacterial presence. Experimental functional validation (e.g., metatranscriptomics) will be necessary to confirm whether these predictions reflect real activity in the field or greenhouse, especially considering the relatively high OOB error of the classification model.

It is important to emphasize that the functional profiles reported in this study were inferred from 16S rRNA amplicon sequencing data using bioinformatic prediction tools and do not constitute direct measurements of gene content or metabolic activity. Accordingly, the identified pathways represent putative functional capacities derived from taxonomic composition rather than experimentally validated gene expression under the applied conditions. These predictions should therefore be interpreted cautiously and warrant further validation through complementary approaches such as shotgun metagenomics, metatranscriptomics, or targeted functional assays.

## 5 Conclusion

Plants treated with WWTP fertilizer (both raw and sterilized) supplemented with strains C1 (*Bacillus pretiosus* CECT30674) and C2 (*Pseudomonas agronomica* CECT30673^T^) exhibited a significant increase in biomass and stem length, together with improved nutritional composition, compared with plants not inoculated with bacteria. Highlighting the role of the C2 strain (*P. agronomica* CECT30673^T^) in its role in promoting plant development in *Q. suber*. Similarly, the incorporation of the strains *B. pretiosus* CECT30674^T^ (C1) and *P. agronomica* CECT30673^T^ (C2) in soils seems to causes a decrease in the minimum inhibitory concentration for all antibiotics commonly used in the clinic. This decrease is particularly significant when examining the most influential variables in the ANOVA analysis and their representation in the corresponding PCA for the cefotaxime and trimethoprim antibiotics, suggesting a possible protective effect against the spread of antibiotic resistance in the environment, which need to be verified in more detailed experiment. Both strains seems to have a strong capacity to adapt to the rhizosphere following inoculation, without causing significant displacement of the native microbial community or altering its functional potential. The sterilization of the matrixes could be an economic issue if the volume of the matrix is very large. For a wide restoration surface should be take into account the cost–benefit about the sterilization. For this reason, the biofertilizer resulting from the addition of the two strains under study to the WWTP residue is postulated for its potential use in the recovery layer of *Q. suber* in degraded soils.

## Data Availability

The datasets presented in this study can be found in online repositories. The names of the repository/repositories and accession number(s) can be found at: https://www.ncbi.nlm.nih.gov/genbank/, PRJNA1153996.
