# Supplementary material for: Recovery of organic waste from a wastewater treatment plant, improved with plant growth promoting bacteria: model of Quercus suber L
Source: Front Microbiol. 2026 May 29;17:1754063. doi: 10.3389/fmicb.2026.1754063 (PMC13260186; doi:10.3389/fmicb.2026.1754063)
Supplement: Supplementary file 1 [file Table_1.docx]

Supplementary material

**Table 1**. MIC (Minimum Inhibitory Concentration). The left column shows the different chemical treatments: W (irrigation with water, WWTP (with biofertilizer) and EDAR_ST (with sterile biofertilizer) and biological C0 (control without inoculum), C1 (Bacillus pretiosus) and C2 (Pseudomonas agronomica). In the top row, you will find the different treatments to which the samples have been subjected. Letter code: AML: amoxicillin; AUG: amoxicillin/ Acid. Clavulanico; CTX: cefotaxime; PP: Piperacillin; CR Ceftriaxone; TZP: Piperacillin/Tazobactam; IMD: Imipenem+EDTA; IMI: imipenem; TS+TZP: Trimethoprim-sulfomethoxazole and piperacillin-tazobactam; NA: nalidixic acid; CIP: ciprofloxacin. Statistically significant differences when p-value < 0,05.

|  | **AML** | **AUG** | **CTX** | **PP** | **CR** | **TZP** | **IMD** | **IMI** | **TS TZP** | **NA** | **CIP** |
| --- | --- | --- | --- | --- | --- | --- | --- | --- | --- | --- | --- |
| **WC0** | 0 | 4 | 8 | 16 | 128 | 6 | 256 | 256 | 75 | 256 | 0,047 |
| **WC1** | 16 | 16 | 16 | 4 | 256 | 8 | 0 | 0 | 8 | 1,5 | 0,25 |
| **WC2** | 6 | 256 | 256 | 256 | 256 | 4 | 0,064 | 0,5 | 4 | 128 | 0,064 |
| **EDARC0** | 256 | 256 | 256 | 3 | 256 | 24 | 0,25 | 8 | 24 | 192 | 0,047 |
| **EDARC1** | 256 | 256 | 256 | 256 | 256 | 48 | 0,19 | 1,5 | 64 | 12 | 0,25 |
| **EDARC2** | 256 | 256 | 256 | 0,96 | 256 | 256 | 0,047 | 8 | 256 | 256 | 0,19 |
| **EDAR_STC0** | 256 | 256 | 256 | 256 | 256 | 32 | 0 | 0 | 24 | 12 | 0,25 |
| **EDAR_STC1** | 256 | 24 | 256 | 64 | 256 | 24 | 0,38 | 0,5 | 16 | 32 | 0,19 |
| **EDAR_STC2** | 256 | 256 | 256 | 256 | 256 | 2 | 0 | 0,5 | 256 | 256 | 0,75 |

**Table 2.** Microbial Metagenomics - Alc Results Report

| **Taxa** | **WC0** | **WC1** | **WC2** | **EDARC0** | **EDARC1** | **EDARC2** | **EDARstC0** | **EDARstC1** | **EDARstC2** |
| --- | --- | --- | --- | --- | --- | --- | --- | --- | --- |
| d__Archaea;p__Crenarchaeota | 0,03 | 0,07 | 0,26667 | 0,03 | 0 | 0,10333 | 0,03667 | 0,10667 | 0,06667 |
| d__Archaea;p__Euryarchaeota | 0 | 0 | 0,10667 | 0 | 0 | 0 | 0 | 0,01 | 0,04 |
| d__Archaea;p__Halobacterota | 0,00667 | 0,16333 | 0,41667 | 0,14 | 0 | 0 | 0,06 | 0,26 | 0,27 |
| d__Archaea;p__Iainarchaeota | 0 | 0 | 0 | 0 | 0 | 0 | 0,00333 | 0 | 0,00333 |
| d__Archaea;p__Micrarchaeota | 0,01667 | 0 | 0 | 0 | 0 | 0 | 0 | 0,03667 | 0 |
| d__Archaea;p__Nanoarchaeota | 0,00333 | 0 | 0 | 0 | 0,03 | 0,05333 | 0,01667 | 0,02333 | 0 |
| d__Archaea;p__Thermoplasmatota | 0,00333 | 0 | 0,00667 | 0,01 | 0 | 0 | 0 | 0,02 | 0,04 |
| d__Bacteria;p__Abditibacteriota | 0,03333 | 0,05333 | 0 | 0,04 | 0,01667 | 0 | 0,00667 | 0,09333 | 0 |
| d__Bacteria;p__Acidobacteriota | 10,14667 | 8,79667 | 7,40667 | 9,68 | 8,54333 | 9,91667 | 9 | 8,01 | 9,41667 |
| d__Bacteria;p__Actinobacteriota | 1,99667 | 2,48 | 2,44667 | 2,86667 | 3,04667 | 2,03 | 1,81667 | 1,21 | 2,2 |
| d__Bacteria;p__Armatimonadota | 0,19667 | 0,49 | 0,42667 | 0,18333 | 0,3 | 0,36333 | 0,20333 | 0,22667 | 0,16 |
| d__Bacteria;p__Bacteroidota | 6,02333 | 7,03667 | 8,49333 | 7,02 | 12,98333 | 6,67667 | 8,91667 | 8,45333 | 9,02333 |
| d__Bacteria;p__Bdellovibrionota | 0,90667 | 0,76 | 0,68 | 0,89333 | 1 | 0,92333 | 1,35667 | 1,02667 | 0,92667 |
| d__Bacteria;p__Chloroflexi | 9,90667 | 7,76 | 8,65333 | 7,20333 | 7,03333 | 9,94333 | 7,67333 | 7,30333 | 8,11 |
| d__Bacteria;p__Cyanobacteria | 0,06 | 0,08 | 0,11 | 0,03333 | 0,01333 | 0,07333 | 0,10667 | 0,06 | 0,05 |
| d__Bacteria;p__Dadabacteria | 0,18333 | 0,03667 | 0,07667 | 0,23333 | 0,07 | 0,09333 | 0,13 | 0,18333 | 0,06333 |
| d__Bacteria;p__Deinococcota | 0 | 0,00667 | 0 | 0 | 0 | 0 | 0,00333 | 0 | 0 |
| d__Bacteria;p__Dependentiae | 0,63667 | 1,19 | 1,29 | 0,77 | 0,66333 | 0,53667 | 1,77667 | 0,83 | 1,05333 |
| d__Bacteria;p__Desulfobacterota | 0,62 | 0,52333 | 0,94667 | 0,65333 | 0,19 | 0,47 | 0,70333 | 0,37 | 0,59 |
| d__Bacteria;p__Elusimicrobiota | 0,11667 | 0,02667 | 0,04 | 0,13 | 0,10333 | 0,03 | 0,14333 | 0,07 | 0,03333 |
| d__Bacteria;p__FCPU426 | 0 | 0 | 0 | 0 | 0 | 0,00333 | 0,00667 | 0,01333 | 0 |
| d__Bacteria;p__Fibrobacterota | 0,11 | 0,12 | 0,38667 | 0,11667 | 0,06333 | 0,17 | 0,13333 | 0 | 0,23333 |
| d__Bacteria;p__Firmicutes | 0,15333 | 4,48 | 1,01667 | 0,20333 | 4,5 | 0,43 | 0,86 | 3,92333 | 0,92667 |
| d__Bacteria;p__Fusobacteriota | 0 | 0,71667 | 0 | 0 | 0,45667 | 0 | 0 | 0,56 | 0 |
| d__Bacteria;p__Gemmatimonadota | 0,47667 | 0,29 | 0,1 | 0,37667 | 0,30667 | 0,41667 | 0,24 | 0,66333 | 0,17667 |
| d__Bacteria;p__Hydrogenedentes | 0,33 | 0,10333 | 0,10333 | 0,05667 | 0,06 | 0,06333 | 0,18 | 0,23667 | 0,13 |
| d__Bacteria;p__Latescibacterota | 0,74333 | 0,41333 | 0,33 | 0,29 | 0,36333 | 0,72667 | 0,27667 | 0,22667 | 0,42333 |
| d__Bacteria;p__MBNT15 | 0,08333 | 0 | 0 | 0,03333 | 0,01667 | 0,03 | 0,01 | 0,01 | 0,02 |
| d__Bacteria;p__Methylomirabilota | 0,01667 | 0 | 0 | 0 | 0 | 0 | 0,00333 | 0,00667 | 0 |
| d__Bacteria;p__Myxococcota | 0,72333 | 0,31333 | 0,23667 | 0,28667 | 0,27667 | 0,28 | 0,39 | 0,68333 | 0,30333 |
| d__Bacteria;p__NB1-j | 0,18667 | 0,05 | 0,11 | 0,35667 | 0,23667 | 0,29333 | 0,21667 | 0,00667 | 0,16 |
| d__Bacteria;p__Nitrospirota | 0,01667 | 0,09667 | 0,02 | 0,13333 | 0,29667 | 0,07667 | 0,08333 | 0,01 | 0,05333 |
| d__Bacteria;p__Patescibacteria | 9,00667 | 11,07667 | 9,52333 | 6,49 | 9,09667 | 11,53333 | 12,88667 | 13,86 | 11,89667 |
| d__Bacteria;p__Planctomycetota | 22,95667 | 24,77667 | 23,46667 | 24,71 | 22,70667 | 24,28667 | 22,99333 | 22,34 | 23,14 |
| d__Bacteria;p__Proteobacteria | 29,58667 | 22,77 | 28,02333 | 28,89 | 20,28 | 25,64667 | 22,82667 | 21,84333 | 25,19 |
| d__Bacteria;p__RCP2-54 | 0,11333 | 0,08333 | 0,19667 | 0,29667 | 0,40667 | 0,13333 | 0,27333 | 0,07333 | 0,31333 |
| d__Bacteria;p__SAR324_clade (Marine_group_B) | 0,29333 | 0,16667 | 0,27333 | 0,30667 | 0,32 | 0,25 | 0,51667 | 0,28 | 0,31667 |
| d__Bacteria;p__Spirochaetota | 0,11667 | 0 | 0,05 | 0,23 | 0,00333 | 0,04333 | 0,07667 | 0,08667 | 0,04 |
| d__Bacteria;p__Sumerlaeota | 0,17 | 0,05333 | 0,05333 | 0,13333 | 0,09667 | 0,10333 | 0,16 | 0,03 | 0,17667 |
| d__Bacteria;p__Verrucomicrobiota | 3,79667 | 4,27667 | 3,98333 | 6,85667 | 6,22333 | 3,26 | 5,41667 | 6,36667 | 3,77667 |
| d__Bacteria;p__WPS-2 | 0,05333 | 0,06 | 0,01 | 0,08333 | 0,03 | 0,01 | 0 | 0,07333 | 0,00333 |
| d__Bacteria;p__WS2 | 0,01333 | 0,17667 | 0,16333 | 0,00667 | 0,07667 | 0,11667 | 0,08333 | 0,07333 | 0,28667 |
| d__Bacteria;p__WS4 | 0 | 0 | 0,08333 | 0,02 | 0 | 0,02667 | 0,12667 | 0,01 | 0,01333 |
| d__Bacteria;p__Zixibacteria | 0,18 | 0,49333 | 0,50333 | 0,23667 | 0,18333 | 0,89667 | 0,29667 | 0,32333 | 0,38 |

**Nutritional parameters measured in leaf**


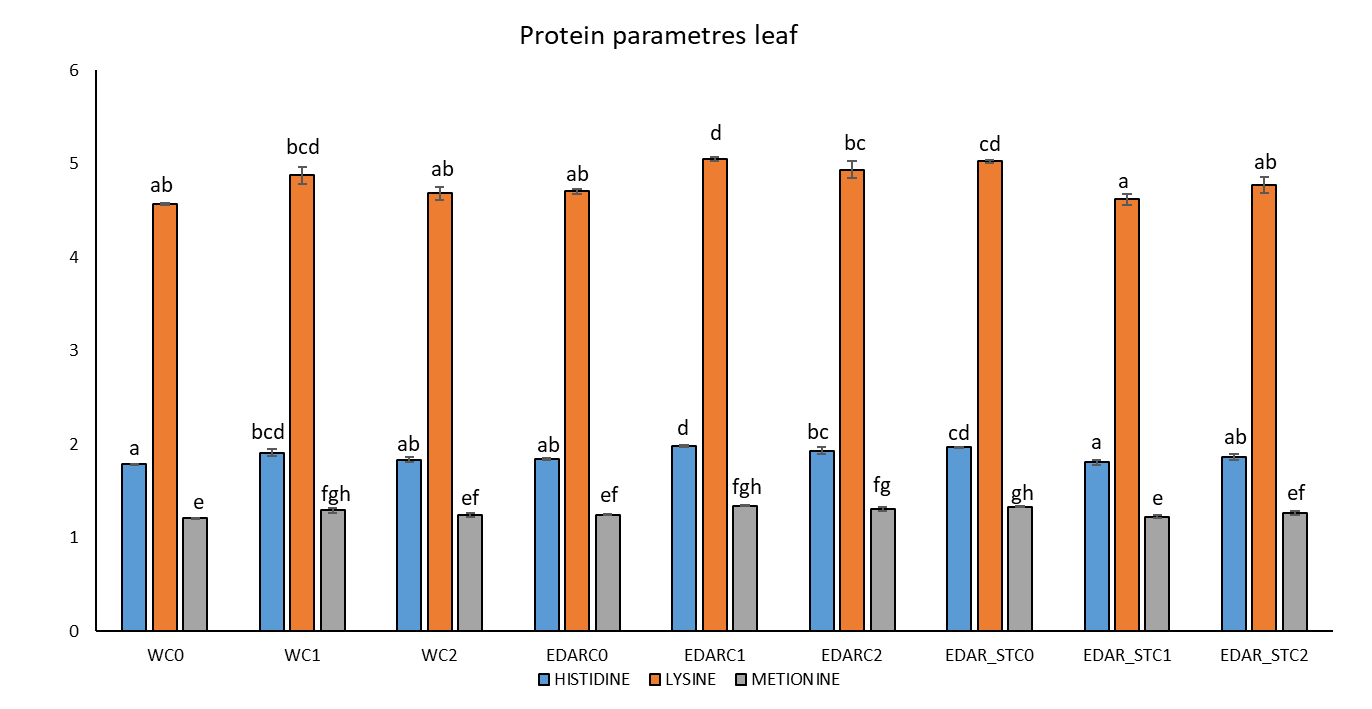


**Figure 1.** *Quercus suber* L. Mean nutritional variables (n=3) related to the aminogram. Bars with identical letters indicate that the average values are not significantly different (p-value < 0,05).


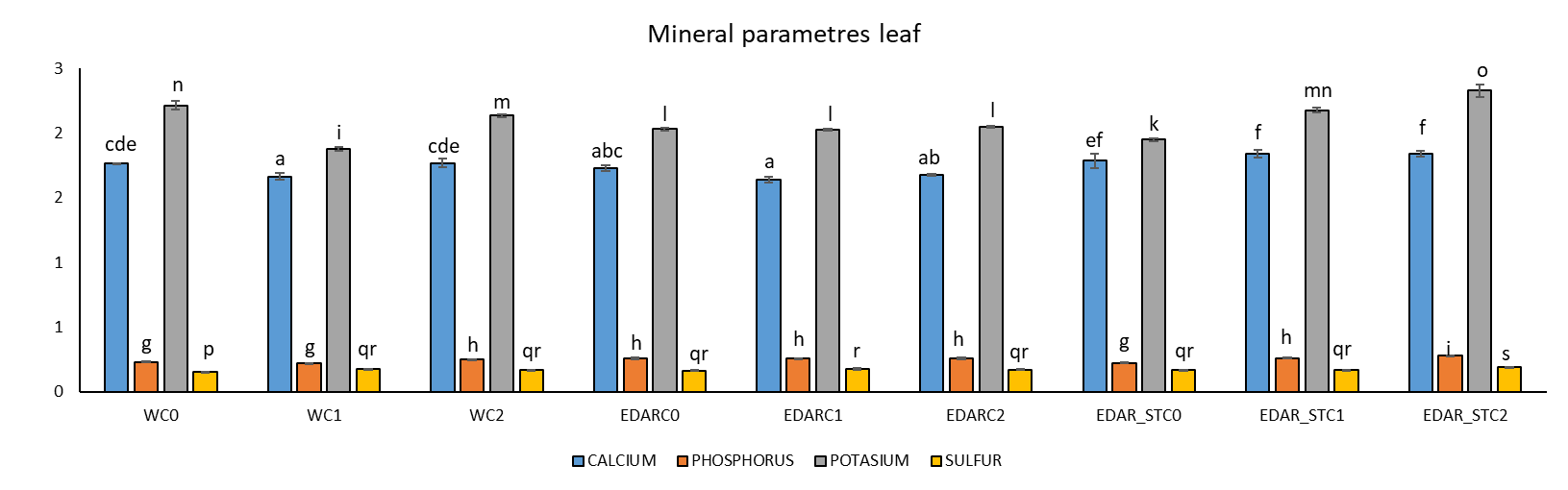


**Figure 2.** *Quercus suber* L. Mean nutritional variables (n=3) related to mineral content. Bars with identical letters indicate that the average values are not significantly different (p-value < 0.05).


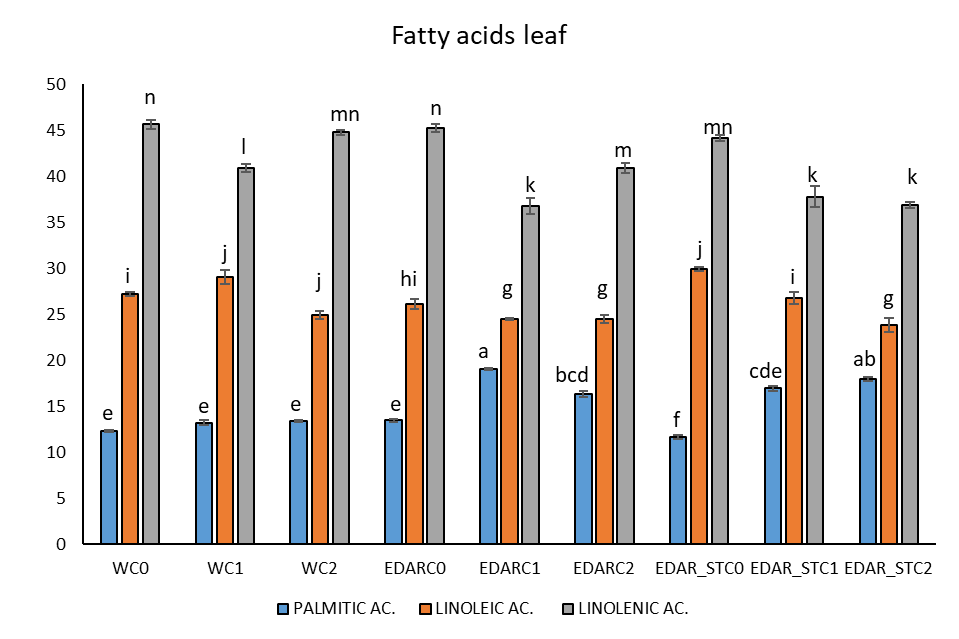


**Figure 3.** *Quercus suber* L. leaf statistics. Mean nutritional variables (n=3) related to fatty acid content. Bars with identical letters indicate that the average values are not significantly different (p-value < 0.05).

**Nutritional parameters measured in stem**

**
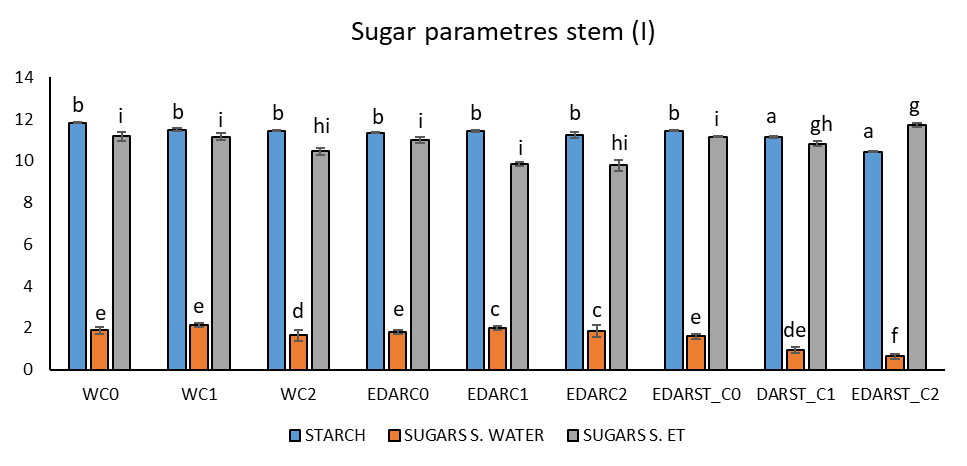
**

**Figure 4.** *Quercus suber* L. stem statistics. Mean nutritional variables (n=3) related to sugar content. Bars with identical letters indicate that the average values are not significantly different (p-value < 0.05).

**
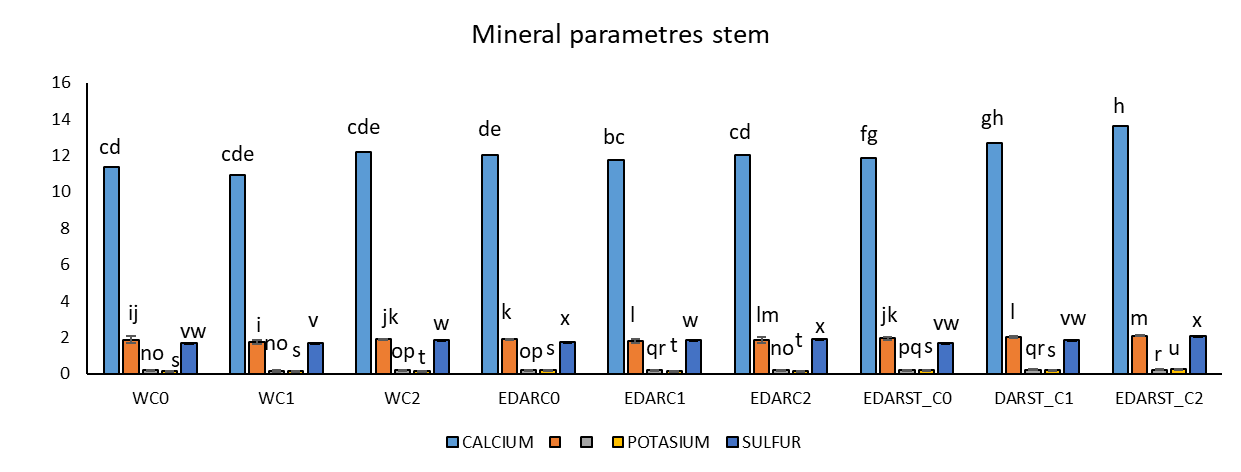
**

**Figure 5.** *Quercus suber* L. stem statistics. Mean nutritional variables (n=3) related to mineral content. Bars with identical letters indicate that the average values are not significantly different (p-value < 0.05).

**
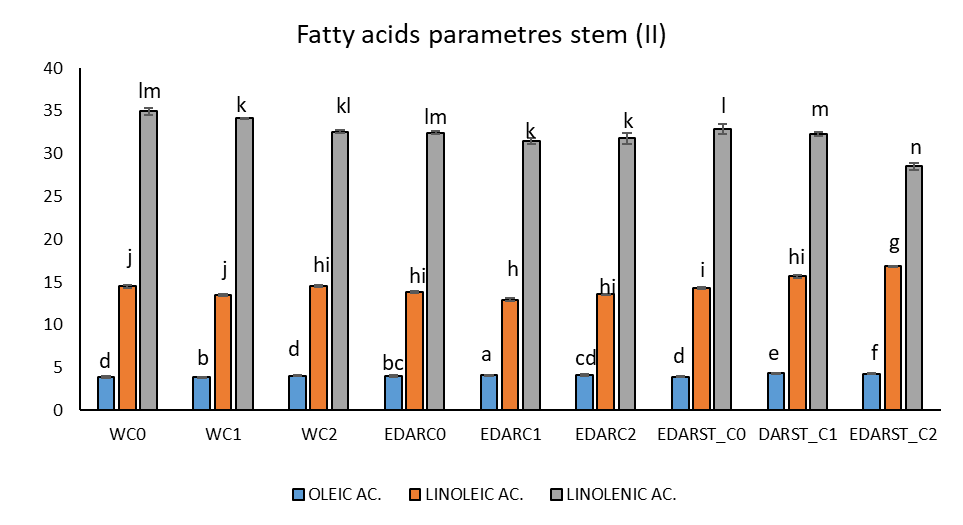
**

**Figure 6.** *Quercus suber* L. leaf statistics. Mean nutritional variables (n=3) related to fatty acid content. Bars with identical letters indicate that the average values are not significantly different (p-value < 0,05).
